# Adjunctive rifampicin to reduce early mortality from *Staphylococcus aureus* bacteraemia (ARREST): study protocol for a randomised controlled trial

**DOI:** 10.1186/1745-6215-13-241

**Published:** 2012-12-18

**Authors:** Guy Thwaites, Cressida Auckland, Gavin Barlow, Richard Cunningham, Gerry Davies, Jonathan Edgeworth, Julia Greig, Susan Hopkins, Dakshika Jeyaratnam, Neil Jenkins, Martin Llewelyn, Sarah Meisner, Emmanuel Nsutebu, Tim Planche, Robert C Read, Matthew Scarborough, Marta Soares, Robert Tilley, M Estée Török, John Williams, Peter Wilson, Sarah Wyllie, A Sarah Walker

**Affiliations:** 1 Department of Infectious Diseases/Centre for Clinical Infection and Diagnostics Research, Kings College London/Guy’s and St. Thomas’ Hospitals NHS Foundation Trust, London, England, United Kingdom; 2 Department of Microbiology, Royal Devon and Exeter NHS Foundation Trust, Exeter, England, United Kingdom; 3 Department of Infection and Tropical Medicine, Hull and East Yorkshire Hospitals NHS Trust, Hull, England, United Kingdom; 4 Department of Microbiology, Plymouth Hospitals NHS Trust, Plymouth, England, United Kingdom; 5 Institutes of Infection and Global Health and Translational Medicine, University of Liverpool, Liverpool, England, United Kingdom; 6 Department of Infection and Tropical Medicine, Royal Liverpool and Broadgreen University Hospitals NHS Trust, Liverpool, England, United Kingdom; 7 Department of Infectious Diseases, Sheffield Teaching Hospitals NHS Foundation Trust, Sheffield, England, United Kingdom; 8 Department of Infectious Diseases and Microbiology, Royal Free London NHS Foundation Trust, London, England, United Kingdom; 9 Department of Medical Microbiology, King’s College Hospital NHS Foundation Trust, London, England, United Kingdom; 10 Department of Infection and Tropical Medicine, Birmingham Heart of England NHS Foundation Trust, Birmingham, England, United Kingdom; 11 Department of Infectious Diseases and Microbiology, Brighton and Sussex University Hospitals NHS Trust, Brighton, England, United Kingdom; 12 Department of Microbiology, Royal United Hospital Bath NHS Trust, Bath, England, United Kingdom; 13 Department of Infectious Diseases and Microbiology, St. Georges Healthcare NHS Trust, London, England, United Kingdom; 14 Department of Infectious Diseases, University Hospitals Southampton NHS Foundation Trust, Southampton General Hospital, Southampton, England, United Kingdom; 15 Department of Infectious Diseases, Oxford University Hospitals NHS Trust, Oxford, England, United Kingdom; 16 Centre for Health Economics, York University, York, England, United Kingdom; 17 Departments of Infectious Diseases and Microbiology, Cambridge University Hospitals NHS Foundation Trust, Cambridge, England, United Kingdom; 18 Department of Infectious Diseases and Microbiology, South Tees Hospitals NHS Foundation Trust, Middlesbrough, England, United Kingdom; 19 Department of Microbiology, University College London Hospitals NHS Foundation Trust, London, England, United Kingdom; 20 Department of Microbiology, Portsmouth Hospitals NHS Trust, Portsmouth, England, United Kingdom; 21 Infections Theme, MRC Clinical Trials Unit, London, United Kingdom; 22 NIHR Biomedical Research Centre, John Radcliffe Hospital, Oxford, United Kingdom

**Keywords:** *Staphylococcus aureus*, Bacteraemia, Rifampicin, Mortality

## Abstract

**Background:**

*Staphylococcus aureus* bacteraemia is a common and serious infection, with an associated mortality of ~25%. Once in the blood, *S. aureus* can disseminate to infect almost any organ, but bones, joints and heart valves are most frequently affected. Despite the infection’s severity, the evidence guiding optimal antibiotic therapy is weak: fewer than 1,500 patients have been included in 16 randomised controlled trials investigating *S. aureus* bacteraemia treatment. It is uncertain which antibiotics are most effective, their route of administration and duration, and whether antibiotic combinations are better than single agents. We hypothesise that adjunctive rifampicin, given in combination with a standard first-line antibiotic, will enhance killing of *S. aureus* early in the treatment course, sterilise infected foci and blood faster, and thereby reduce the risk of dissemination, metastatic infection and death. Our aim is to determine whether adjunctive rifampicin reduces all-cause mortality within 14 days and bacteriological failure or death within 12 weeks from randomisation.

**Methods:**

We will perform a parallel group, randomised (1:1), blinded, placebo-controlled trial in NHS hospitals across the UK. Adults (≥18 years) with *S. aureus* (meticillin-susceptible or resistant) grown from at least one blood culture who have received ≤96 h of active antibiotic therapy for the current infection and do not have contraindications to the use of rifampicin will be eligible for inclusion. Participants will be randomised to adjunctive rifampicin (600-900mg/day; orally or intravenously) or placebo for the first 14 days of therapy in combination with standard single-agent antibiotic therapy. The co-primary outcome measures will be all-cause mortality up to 14 days from randomisation and bacteriological failure/death (all-cause) up to 12 weeks from randomisation. 940 patients will be recruited, providing >80% power to detect 45% and 30% reductions in the two co-primary endpoints of death by 14 days and bacteriological failure/death by 12 weeks respectively.

**Discussion:**

This pragmatic trial addresses the long-standing hypothesis that adjunctive rifampicin improves outcome from *S. aureus* bacteraemia through enhanced early bacterial killing. If proven correct, it will provide a paradigm through which further improvements in outcome from *S. aureus* bacteraemia can be explored.

**Trial registration:**

Current Controlled Trial ISRCTN 37666216

## Background

*Staphylococcus aureus* bacteraemia (SAB) is one of the most common serious bacterial infections worldwide. In the UK alone there are more than 12,000 cases of SAB each year and around 25% of these patients die [1,2]. Once *S. aureus* has entered the bloodstream it can disseminate to cause metastatic infection of almost any organ in the body. The sites most commonly affected are the heart valves (5-10% of cases), joints (5% of cases), intervertebral discs (5% of cases), bones (2% of cases) and, less commonly, the brain, spleen and kidney. Implanted prosthetic material, such as artificial heart valves or joints, is at especially high risk of becoming infected.

Current treatment guidelines recommend that SAB should be treated with at least 14 days of an intravenous (IV) beta-lactam antibiotic, or a glycopeptide if the bacteria are meticillin-resistant. Combination antimicrobial therapy is generally not recommended, except in severe meticillin-resistant *S. aureus* (MRSA) infections (e.g. endocarditis, prosthetic joint infections) [3-6]. However, the evidence supporting these recommendations is weak: fewer than 1,500 patients have been entered in 16 randomised controlled trials (RCT) of SAB antimicrobial therapy published over the last 50 years [7]. Most of the recommendations are based on uncontrolled observational studies and clinical experience, and views of how to manage SAB differ widely [8,9].

### How might adjunctive rifampicin improve outcome from *S. aureus* bacteraemia?

The best clinical predictor of complications and death from SAB is the persistence of bacteria in blood 48–96 h after the start of active antimicrobial therapy [10-12]. Persistent bacteraemia (>48 h) occurs in around 40% of patients, despite prompt removal of any infected focus and effective antimicrobial therapy [10,11], and increases the patient’s risk of metastatic complications and death nearly five-fold [10]. Why *S. aureus* persists in blood despite treatment with antibiotics with good *in vitro* activity is uncertain, but is probably explained by the failure of currently recommended first-line antibiotics (beta-lactams and glycopeptides) to kill bacteria associated with pus (dead or dying neutrophils), viable cells or biofilms. The well-documented survival of *S. aureus* within each of these ecological niches may lead to persistent bacterial seeding of the bloodstream and recurrent, recalcitrant infection. In addition, we have recently proposed that bloodstream neutrophils may act as “Trojan horses” for *S. aureus* dissemination, providing bacteria with further protection from first-line antibiotics with poor intracellular activity such as the recommended beta-lactams and glycopeptides [13].

Three properties make rifampicin an attractive, if unproven, adjuvant antibiotic for SAB treatment. First, it has good oral bioavailability [14]. Second, it penetrates cells [15,16], tissues and biofilms [17,18] better than beta-lactam and glycopeptide antibiotics [19,20] (the current mainstays of SAB treatment) and, therefore, in combination with these agents, may resolve serious *S. aureus* infections faster and more effectively [18]. Third, it is cheap: a daily 600-mg dose costs £0.73 by mouth and £7.67 intravenously [21]. Yet, despite all these properties, the potential advantages of adjunctive rifampicin for the treatment of severe *S. aureus* infections in humans remain theoretical. There are insufficient data from only 246 patients randomised between rifampicin vs. non-rifampicin containing regimens in controlled trials to confirm or refute a beneficial effect.

### What are the potential problems of using adjunctive rifampicin for *S. aureus* bacteraemia?

There are three important potential problems with using rifampicin for the treatment of SAB: the development of rifampicin-resistant bacteria, interactions with other drugs and hepatic toxicity. Resistance can be acquired rapidly when rifampicin is used alone in treatment, resulting from mutations in the drug’s binding site (the β-subunit of the bacterial DNA-dependent RNA polymerase). The frequency with which rifampicin resistance develops during the combination therapy of SAB is difficult to assess from the published literature, varying from 0/433 patients treated with adjunctive rifampicin in three non-randomised studies of serious *S. aureus* infections [22-24] to 20-40% of patients in other smaller case series [25-27]. Interactions with other drugs are mediated by rifampicin’s ability to increase their metabolism through the potent induction of the hepatic cytochrome p450 system, but how frequently this influences treatment outcomes is uncertain. Lastly, rifampicin can cause hepatic toxicity, although the enormous worldwide experience of using rifampicin for the prevention and 6-month treatment of tuberculosis confirms the drug is extremely well tolerated and causes clinically significant hepatitis in <1% of patients [28].

### Clinical evidence for using adjunctive rifampicin for *S. aureus* bacteraemia

**Figure 1:**
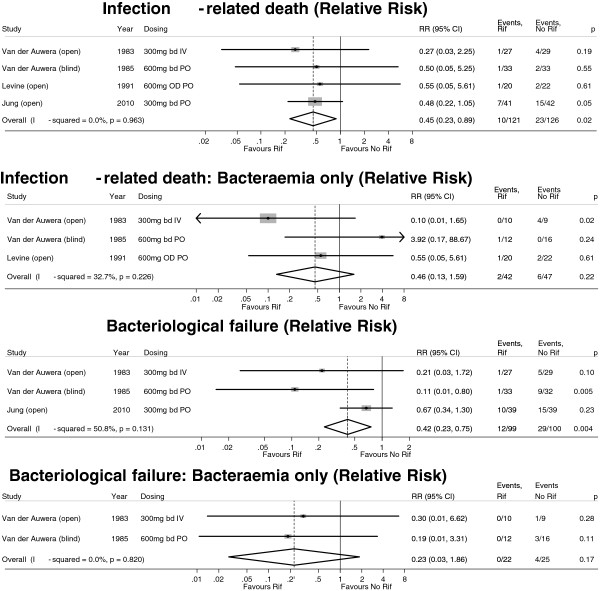
**Meta-analysis (fixed effects) of four trials of adjunctive rifampicin for severe *S. aureus *disease, including bacteraemia.**

Four randomised controlled trials, involving 246 patients in total, have examined the effectiveness of adjunctive rifampicin for serious *S. aureus* infections, including patients with bacteraemia [29-32]. The first two trials, published more than 25 years ago, enrolled adults with any serious *S. aureus* infection, of whom 47/121 (39%) were bacteraemic at randomisation [29,33]. The third trial enrolled 42 adults, all with SAB and endocarditis [31], and the fourth enrolled 83 adults admitted to an intensive care unit with MRSA pneumonia; only 9/83 (11%) were bacteraemic [32]. We performed a stratified meta-analysis of the results from these trials (Figure 1); subgroup analysis of bacteraemic adults was possible for all but the fourth trial, which did not provide sufficient data. Overall, adjunctive rifampicin reduced infection-related deaths by 55% (*p* = 0.02) and bacteriological failure by 58% (*p* = 0.004), with similar (54%, 77%) but non-significant (*p* = 0.22, *p* = 0.17) reductions in the bacteraemic subgroup (*n* = 89).

The daily dose of rifampicin in these trials varied from 600 mg to 1,200 mg. Significant drug interactions were not reported in any of the trials, and details concerning hepatic toxicity were not provided in the first three trials. The most recent trial reported 6/41 (15%) patients treated with rifampicin developed hyperbilirubinaemia (compared to 1 control patient) but the impact on treatment was not described. This trial was also the only one to report rifampicin resistance developing on treatment: new resistance was found in 14/41 (34%) rifampicin-treated patients, although it did not appear to have a significant impact on clinical cure rates [32].

There are limited data from uncontrolled, observational studies supporting the use of adjunctive rifampicin, although, given the potential for confounding by indication, their results must be interpreted cautiously. A prospective study of 381 adults with SAB found the mortality of those with severe disease was halved in those who received adjunctive rifampicin (mortality 38% vs. 17%, *p* < 0.001), without an increased incidence of rifampicin resistance [23]. A recent retrospective analysis of patients with staphylococcal sternal wound infections, 35% of whom had SAB, reported adjunctive rifampicin was independently associated with a reduced risk of treatment failure (hazard ratio 0.26, 95% CI 0.10–0.64, *p* = 0.004) [24].

Current USA and UK guidelines only recommend adjunctive rifampicin for the treatment of severe MRSA infections, specifically endocarditis, bone and joint infections, and infections involving prostheses (category II evidence) [4,6]. But with weak support for these recommendations it is unsurprising few UK physicians follow them in practice [34]. In short, currently available evidence suggests adjunctive rifampicin may substantially improve the outcome from SAB but there are too few data to balance the potential risks against the benefits. We will, therefore, perform a large, multicentre, randomised controlled trial with the aim of providing a definitive answer to the question, ‘Does adjunctive rifampicin improve outcome from SAB?’

## Methods

### Study hypothesis and objectives

We hypothesise that adjunctive rifampicin will enhance killing of *S. aureus* early in the course of antibiotic treatment, sterilise infected foci and blood faster, and thereby reduce the risk of dissemination, metastatic infection and death. Therefore, the primary objective of the trial is to investigate the impact of adjunctive rifampicin on both all-cause mortality through 14 days from randomisation and bacteriological failure or death through 12 weeks from randomisation.

The trial’s secondary objectives include:

• assessing toxicity and emergence of resistance associated with adjunctive rifampicin

• identifying the duration of bacteraemia and potential mechanisms of action of rifampicin through pharmacokinetic/pharmacodynamic (PK/PD) investigations

• assessing the cost effectiveness of the use of adjunctive rifampicin and other antibiotic therapy for SAB in the UK NHS.

### Study setting

The study will be performed in 17 large UK NHS Hospital Trusts: Guy’s and St. Thomas’ Hospitals NHS Foundation Trust; Oxford University Hospitals NHS Trust; University College London Hospital NHS Foundation Trust; Royal Free Hampstead NHS Trust; Kings College Hospital NHS Foundation Trust; Brighton and Sussex University NHS Trust; Royal Liverpool and Broadgreen University Hospitals NHS Trust; Sheffield Teaching Hospitals NHS Foundation Trust; Cambridge University Hospitals NHS Foundation Trust; Royal United Hospital Bath NHS Trust; Royal Devon and Exeter NHS Foundation Trust; Plymouth Hospitals NHS Trust; Hull and East Yorkshire Hospitals NHS Trust; South Tees Hospitals NHS Trust; Birmingham Heart of England NHS Foundation Trust; St. Georges Healthcare NHS Trust; and Portsmouth Hospitals NHS Trust. Additional NHS centres may be added after the start of the trial, if required.

### Patient selection

Patients will be considered eligible for enrolment in this trial if they fulfil all the inclusion criteria and none of the exclusion criteria as defined below. Patients will be identified through the clinical microbiology laboratory and the infectious diseases/microbiology consult service of each centre. All the trial centres run a clinical consult service for all cases of SAB and identify such patients as soon as their blood cultures become positive. The infectious diseases physicians and microbiologists responsible for this service will alert their colleagues responsible for trial recruitment and they will arrange for the patient to be seen and assessed for eligibility. All eligible patients will be hospital inpatients. When possible, patients will be screened for eligibility on the day their blood cultures flags positive with *S. aureus*; this usually takes 24–48 h from inoculation of the culture bottle.

### Inclusion criteria

1. Adults (18 years or older)

2. *S. aureus* (meticillin-susceptible or resistant) grown from at least one blood culture

3. Less than 96 h of active antibiotic therapy for the current infection, not including rifampicin

4. Patient or legal representative (LR) provides written informed consent

### Exclusion criteria

1. Infection not caused by *S. aureus* alone in the opinion of the infection specialist (e.g. *S. aureus* is considered a blood culture contaminant or polymicrobial culture with another organism likely to be contributing clinically to the current infection)

2. Sensitivity results already available and demonstrate rifampicin-resistant *S. aureus* (defined by British Society for Antimicrobial Chemotherapy *in vitro* disc susceptibility testing)

3. Infection specialist, in consultation with the treating physician, considers rifampicin is contraindicated for any reason

4. Infection specialist, in consultation with the treating physician, considers rifampicin treatment is mandatory for any reason

5. Infection specialist suspects active infection with *Mycobacterium tuberculosis*

6. Previously been randomised in ARREST for a prior episode of SAB

### Randomisation

Eligibility will be confirmed by a web-based programme and patients randomised to two parallel groups in a 1:1 ratio: standard intravenous antibiotic therapy plus 14 days placebo or standard intravenous antibiotic therapy plus 14 days rifampicin. Randomisation will be stratified by clinical site, as blinded drug (in fully made-up and labelled treatment packs) will be pre-shipped to local pharmacies. Randomisation lists will be computer-generated based on random permuted blocks. A 24-h web-based randomisation service will be provided.

### Treatment of patients

**Figure 2:**
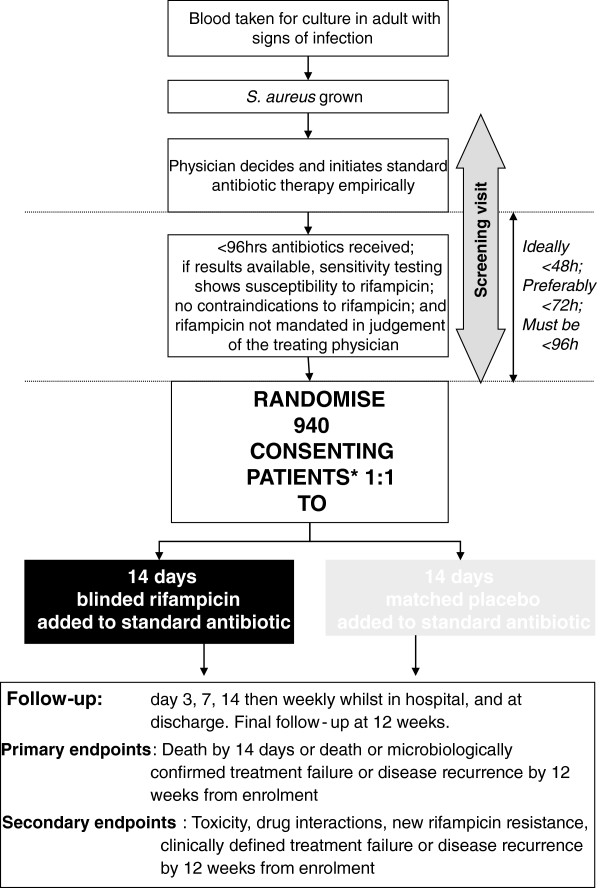
Flow of participants through the trial.

All patients will receive the standard of care antibiotic to treat SAB that they would have received if they had not been enrolled in the ARREST trial. In addition, patients will be randomised to receive rifampicin or placebo (investigational medicinal product) as an extra medication for 2 weeks (Figure 2). Rifampicin/placebo for 14 days will be dispensed at randomisation from the site pharmacy in oral (capsule) or intravenous formulations, according to the attending physician’s preference and the patient’s status. Rifampicin (300-mg capsules; Sanofi Aventis Ltd., Surrey, UK) will be over-encapsulated to make them indistinguishable from placebo. However, rifampicin for intravenous infusion comes as a vial of red powder that requires reconstitution with 10 ml of water for infusion with saline. The resulting fluid for intravenous infusion is orange. It is impossible to safely and reliably produce a red-powder placebo that will produce an identical orange infusion. Therefore, we accept that the nurses making up the intravenous drug for the infusion will not remain blind to the treatment. They will be instructed not to divulge the colour of the drug to the physicians caring for the patient. In addition, the infusion will be covered by an opaque bag to disguise the treatment.

The dose of rifampicin/placebo will be prescribed according to the patient’s weight:

• those <60 kg will receive 600 mg every 24 h

• those >60 kg will receive 900 mg every 24 h

Oral rifampicin/placebo may be given once or twice daily, according to physician preference. If 900 mg is given twice daily, doses will be unequal (600 mg a.m., 300 mg p.m.). The study treatment will be given for 14 days, unless fewer than 14 days of standard antibiotic therapy is planned, in which case rifampicin/placebo will be given until standard antibiotic treatment ends.

The choice and duration of the standard antibiotic therapy that accompanies the rifampicin/placebo will be left to the attending physician, but is expected to be either a beta-lactam (e.g. flucloxacillin) or a glycopeptide (vancomycin or teicoplanin). Daptomycin and linezolid are the other alternatives, although current data from the trial sites indicate <1% receive daptomycin and around 5% receive linezolid as initial first-line therapy. A pre-specified subgroup analysis will be conducted by standard antibiotic therapy, at a class level, and according to individual drugs where these are used by >10% of the trial population. If the physician wishes to use other antibiotics (or rifampicin) after 14 days (the study drug duration), this will be open-label, but recorded.

### Blinding issues

Rifampicin can turn urine (and tears/sweat) reddish-orange. It is impossible to safely replicate this effect with a placebo; therefore urine discolouration will be a potential source of unblinding, particularly of the patient. There is, however, considerable inter- and intra-individual variability in rifampicin’s effect on urine colour. In some patients, the colouration is slight and can be hard to distinguish from the dark, concentrated urine frequently observed in acutely unwell patients. In many, the orange urine colour becomes less marked over time. In addition, the opportunity for physicians to examine the urine at the bedside will only occur in patients with urinary catheters. Catheters will not be required by all patients and are usually removed at the earliest opportunity. We will also limit the opportunity for physicians to inspect urine by ensuring the catheter bags are emptied regularly and urine it is not allowed to accumulate in large volumes.

### Assessments and follow-up

All participants will be followed by the site study teams for 12 weeks for evaluation of all-cause mortality, morbidity and toxicity. Subsequent follow-up will be electronic through hospital records (consent will be sought for this together with consent for trial participation). To assess the outcome measures, patients will be visited on the ward by the site PI, one of their clinical team (e.g. Specialist Registrar) or a research nurse on day 3, 7, 10 and 14 and then weekly whilst still in hospital through 12 weeks (see Table 1). All data will be recorded on (electronic) eCRFs.

**Table 1:** The ARREST trial assessment schedule

	**Day**											
	**Screening**	**0**	**1**	**2**	**3**	**5**	**7**	**10**	**14**	**Weekly until discharged**	**84**	**Potential failure/recurrence**
**All patients**												
Eligibility assessment	X											
Patient information sheet and consent	X											
Randomisation		X										
Clinical assessment^(a)^		X			X		X	X	X	X	X	X
Resource utilisation^(b)^		X					X		X	X	X	
EQ-5D		X					X		X		X	X
Blood culture with sensitivities (10 ml)^(c)^	(X)	X			X		X					X
EDTA blood^(d)^ (1.5 ml)		X										
Clotted blood^(e)^ (5 ml)												
CRP	(X)	X			X			X	X			X
ALT, ALP, bilirubin	(X)				X			X				
Serum storage		X			X				X			
Whole blood^(f)^ (5 ml)		X										
**Subset of patients recruited to the intensive PK/PD substudy**^**(g)**^												
Lithium heparin blood (3 × 3 ml/ time point) for antibiotic concentration assays^(h)^		X			X		X					
Lithium heparin blood (10 ml) for compartment studies^(i)^		X	X	X	X	X	X	X	X			
Clotted blood^(e)^ (5 ml)												
CRP		X	X	X	X	X	X	X	X			X
ALT, ALP, bilirubin					X			X				
Creatinine		X			X			X				
Serum storage		X	X	X	X	X	X	X	X			X
Blood culture (10 ml)		X	X	X	X	X	X	X	X			X
**Subset of patients recruited to the sparse PK/PD substudy**^**(j)**^												
Lithium heparin blood (3 × 3 ml in total) for antibiotic concentration assays^(j)^		X			X							
Clotted blood^(e) ^(5 ml)												
CRP		X			X			X	X			X
ALT, ALP, bilirubin				X			X					
Creatinine		X										
Serum storage		X			X				X			X
Blood culture (10 ml)		X	X	X	X		X					X

() indicates tests that will have already been performed as part of standard management.

*If a patient has already been discharged from hospital before day 7, 10 or 14, additional investigations requiring a blood draw (culture, CRP, ALT, ALP, bilirubin, serum storage) are not required (patients should not be asked to attend outpatient appointments on these days, but to return at 12 weeks only).

(a) Including likely source and focus of infection, co-morbidities, duration of symptoms, temperature and record of concomitant medications (including all non-study antibiotics) at enrolment. Follow-up assessments will record new symptoms and signs indicating secondary site infections, all surgical interventions performed to treat the disease, grade 3 or 4 or serious adverse events, adverse events of any grade leading to modification of rifampicin/placebo dose or interruption/early discontinuation, any drug interactions leading to dose modification of any drug (including concomitant medications) and all changes in antimicrobial prescribing.

(b) Resources used whilst in hospital will be recorded by health-care professionals (or any assigned representatives). These will include days spent in wards, procedures or laboratory tests undertaken and concomitant medication. After discharge, resource use will be self-reported by the patient. A data collection form will be developed that records post-discharge re-hospitalisations and contact with clinicians (hospital, GP, etc.).

(c) Blood cultures will have already been taken prior to the screening assessment from which the potential *S. aureus* bacteraemia will have been identified. *S. aureus* isolated from blood cultures taken on days 0, 3 and 7 must have at least rifampicin susceptibility tests performed in order to evaluate the secondary endpoint acquisition of rifampicin resistance, although typically the routine panel of antimicrobial sensitivities will be performed (all susceptibilities will be recorded). All *S. aureus* isolates to be stored locally, then shipped annually to central storage facility in Oxford and Brighton for biobanking. All repeat isolates will be genotyped to define relapse or re-infection. Blood cultures may be taken at any other time points necessary for clinical management of the patient. If these are considered to reflect potential failure of treatment or recurrence, then sensitivities should be performed as above. Results of any additional blood cultures done should be recorded on ARREST CRFs, and *S. aureus* isolates stored and rifampicin susceptibility tested.

(d) For measurement of haemoglobin, white cell count, lymphocytes, neutrophils, platelets.

(e) For measurement of C-reactive protein (CRP), alanine transaminase (ALT), bilirubin and alkaline phosphatase (ALP) at time points shown. Serum creatinine will only be measured in the PK/PD substudies on days 0, 3, and 10; 2 ml of serum will be saved from clotted blood taken as shown, stored locally, then shipped to a central archive at King’s College London. CRP and liver function tests are routine investigations for patients with suspected *S. aureus* bacteraemia, and results of pre-screening investigations should also be recorded on the screening CRF.

(f) 2.5 ml into EDTA and 2.5 ml into PAXgene blood RNA tube (Qiagen). Store EDTA blood for later DNA extraction and PAXgene tube for later RNA extraction. Samples will be stored locally before shipping to King’s College London for DNA/RNA extraction and archiving if the patient has consented for human DNA/RNA storage.

(g) Only patients enrolled at Guy’s and St. Thomas’, University College London and Addenbrookes Hospitals will be approached.

(h) A total of 9 × 3-ml blood samples will be taken for drug concentration assays. Aliquots of plasma will be rapidly frozen at -70^0^C and stored locally before shipment in batches to Liverpool for subsequent measurement of antibiotic concentrations. Day 0 samples will be taken after the initial dose of study drug.

(i) 10 ml of blood will be taken and centrifuged over fycol to separate the blood into plasma, peripheral blood mononuclear cells and neutrophil fractions. Each fraction will be cultured and cells will be frozen for later characterisation.

(j) A total of 3 × 3-ml blood samples will be taken for drug concentration assays. Only patients enrolled at Oxford, Liverpool and Brighton will be approached. Day 0 samples will be taken after the initial dose of study drug.

### Outcome measures

The trial’s co-primary outcomes will be:

• all-cause mortality up to 14 days

• death or microbiologically confirmed treatment failure or disease recurrence (bacteriological failure) up to 12 weeks from randomisation.

We have chosen these co-primary outcome measures as they are both severe events and are thus unlikely to be influenced by unintended unmasking of treatment allocation (for example, through rifampicin discolouring urine) and, if significantly reduced by rifampicin, will provide an unequivocal stimulus to change practice.

Microbiologically confirmed treatment failure will be defined as symptoms and signs of infection for longer than 14 days from randomisation with the isolation of same strain of *S. aureus* (confirmed by genotyping) from a sterile site (e.g. blood, joint fluid, pus from tissue). Disease recurrence will be defined as the isolation of the same strain of *S. aureus* from a sterile site after at least 7 days of apparent clinical improvement. The same strain will be defined as one with the same genotype by multi-locus sequence [35] and spa-typing [36].

The secondary outcome measures will be:

• death or clinically defined treatment failure or disease recurrence by 12 weeks (clinical failure being assessed by an independent endpoint review committee blind to the treatment allocation)

• duration of bacteraemia (blood cultures will be taken on days 3 and 7 following randomisation)

• development of rifampicin-resistant *S. aureus*

• grade 3/4 adverse events

• serious adverse events

• modification of any treatment (including concomitant medications) due to drug interactions.

• compliance with blinded rifampicin/placebo

• health-care-related costs of *S. aureus* bacteraemia

• EuroQol-5D questionnaire (EQ-5D)

Cause of death, microbiological and treatment failure/recurrence will be adjudicated by an Endpoint Review Committee (ERC) blinded to randomised allocations.

### Safety

Rifampicin is given to around 9 million people each year as the first-line, 6-month treatment of tuberculosis. It is well tolerated and there is extensive experience amongst all the trial centres investigators (infectious diseases physicians and microbiologists) of its safe use. Hepatitis is the most important side effect of rifampicin: asymptomatic rises in liver transaminases occur in around 10% of patients taking rifampicin and requires no action other than careful monitoring. Significant rifampicin-induced hepatitis [transaminases >5× the upper limit of normal (ULN) +/− symptoms] is rare (<1% of patients) and usually resolves with discontinuation of the drug. The liver function tests of patients will be routinely monitored twice whilst taking the study drug (day 3 and day 10), which is more frequent than recommended by UK guidelines when using rifampicin to treat tuberculosis [37]. However, the trial patients are likely to be more acutely unwell than patients with tuberculosis and may require closer monitoring; additional tests may be requested at any time where necessary for patient management. Physicians will stop blinded rifampicin/placebo (without unblinding) if in their opinion potentially rifampicin-related severe hepatic toxicity occurs. Potentially fatal hepatic injury is extremely unlikely given the relatively short course of rifampicin, laboratory monitoring and early withdrawal if the transaminases rise >5× the upper limit of normal. The commonest side effect of rifampicin is orange urine, but this effect is completely harmless. Rifampicin also colours tears orange and can stain contact lenses.

Rifampicin induces the hepatic metabolism of many other drugs, which can result in their sub-therapeutic concentrations. The recruiting infection specialists will be responsible for identifying clinically important interactions and ensuring appropriate action is taken to reduce the risks to patients; this may include judging patients receiving these medications as not eligible to join ARREST.

All such adverse events will be reported on CRFs, together with adverse events of any grade leading to modification of the rifampicin/placebo dose or its interruption/early discontinuation. All SAEs and SARs should be notified to the MRC CTU within 24 h of the investigator becoming aware of the event.

### Sample size

Our current observational study data indicate 16% and 24% of all cases of SAB die by 14 days and 12 weeks respectively. Data from Oxford (personal communications) suggest that a further 10% of patients have repeat isolation of *S. aureus* over the 12 weeks following initial bacteraemia. Assuming 80% power, two-sided alpha 0.025 (to adjust for multiple testing given 2 co-primary endpoints) and a 10% loss to follow-up by 12 weeks, we would need to randomise 920 patients to detect a 30% relative reduction in bacteriological failure/death from 35% to 25%, an absolute difference of 10% corresponding to an number needed to treat (NNT) of 10. The meta-analysis of RCT data (Figure 1) suggests adjunctive rifampicin reduced infection-attributable death by around 50% in all patients with serious *S. aureus* infections (relative risk 0.45, 95% CI 0.23 to 0.89), with a similar, albeit non-significant, effect in the subgroup with bacteraemia (relative risk 0.46, 95% CI 0.13-1.59). These findings strongly support a large effect size (40-50% relative reduction) in mortality. Assuming 80% power, two-sided alpha 0.025 and a lower 4% loss to follow-up by 14 days (as most patients will remain in hospital), we would need to randomise 940 patients to detect a 45% relative reduction in mortality from 16% to 9%, an absolute difference of 7% and a NNT of 14. The total sample size is therefore 940 patients. This provides 68% and 57% power to detect smaller relative differences of 25% and 35% in bacteriological failure/death and death respectively (other assumptions as above, alpha = 0.025). All statistical tests of association in the analyses will be interpreted with respect to a 0.025 rather than 0.05 threshold.

### Statistical analysis

Rifampicin is hypothesised to be superior to standard of care, and therefore the proposed analysis will be by intention to treat, including all randomised patients with all participants analysed according to the study group to which they were randomised regardless of subsequent treatment received. The co-primary endpoints (all-cause mortality through 14 days, bacteriological failure/death through 12 weeks) will be compared using time-to-event methods (Kaplan-Meier plots and log-rank tests, Cox proportional hazards regression), as will time to clinical treatment failure/bacteriological failure/death. Duration of bacteraemia will be compared using interval-censored time-to-event methods. Patients not completing follow-up and not known to have died will be censored at their last contact in the primary analyses; sensitivity analyses will assume all such patients were alive at 12 weeks (i.e. assuming that vital status tracing is robust and reliable). Primary analysis will not stratify for site, as there may be some sites with no events that would therefore not contribute to treatment comparisons; secondary analysis will be conducted stratified by site. Primary analysis will include all randomised patients: secondary analysis will exclude those (expected <1%) who are subsequently identified as having rifampicin-resistant *S. aureus* on susceptibility testing. The frequency of serious, grade 3 and 4, and drug-modifying adverse events will be tabulated by body systems and by randomised groups, and the groups will be compared using Fisher’s exact test.

Patients enrolled in the trial are likely to have heterogeneous underlying conditions (e.g. cardiac, dialysis, cancer). However, precisely because the SAB will have been acquired in addition to any underlying condition, there is no a priori reason why rifampicin should be more or less effective in any comorbid subgroup, other than as a consequence of drug interactions with concomitant medications, which will be assessed as a secondary endpoint. Planned subgroups analyses will therefore include time from initiation of antibiotics to initiation of randomised treatment, time from randomisation to initiation of randomised treatment, intended oral randomised treatment frequency (once vs. twice daily), initial treatment with oral study drug only or regimen containing IV study drug, class of primary antibiotic treatment, other antibiotic adjuncts (e.g. gentamicin), MRSA/MSSA, IV catheter-associated infection/other, deep focus/no deep focus, endocarditis/no endocarditis and age. An additional subgroup analysis will be conducted splitting participants into terciles of baseline C-reactive protein (CRP).

### Cost-effectiveness analysis

We will conduct a cost-effectiveness analysis of therapy for SAB in the NHS. This analysis will be informed by the results of the trial. Due to the limited trial follow-up period and the possibility of existing treatment options other than those in the trial, we propose a framework based on decision analysis. Such an approach allows including findings from the trial in the context of the existing evidence on all treatments of interest and is the preferred approach for societal decision making in the UK.

### Ethical issues

The trial complies with the principles of the Declaration of Helsinki (2008). Written, informed consent will be obtained from all patients, or their legal representatives (LRs) if they lack capacity, before enrolment by the site PI or an appropriately trained Consultant, Specialist Registrar or Research Nurse. Patients (or their LRs) would be free to withdraw from the trial at any time, and this will be explicitly stated in patient information sheets.

Incapacitated adults may be included in the trial as we consider many of these adults will have the most severe infection and therefore represent the group that might stand most to gain from the enhanced anti-microbial activity of rifampicin. We anticipate around 10% of patients with *S. aureus* bacteraemia will be critically ill and incapacitated on intensive care. In this circumstance, the site PI or another experienced and independent physician will follow the UK Mental Capacity Act (2005) to formally assess the capacity of the individual to make an informed decision to participate in the trial. Once incapacity has been confirmed written informed consent will be sought from either a personal (e.g. a relative) or a nominated LR (e.g. Consultant Intensivist caring for the patient, but not involved in the trial). If the subject regains capacity during treatment they will be informed of the consent given by their LR and their wishes respected concerning on-going participation. If they are happy to remain in the trial, the patient should complete a patient consent form at this time.

The trial protocol (V1.02) received national research ethics committee approval in April 2012. Clinical Trials Authorisation was granted by the Medicines and Healthcare products Regulatory Agency (MHRA) in May 2012.

### Patient and public involvement

The ARREST trial has been developed with the Healthcare-associated Infection Service Users Research Forum (SURF: http://www.hcaisurf.org). A member of SURF (Jennifer Bostock) will represent patients and the public on the ARREST Trial Steering Committee. Ms Bostock has advised on the inclusion of incapacitated adults and the application of the Mental Capacity Act, and the information provided to patients. SURF will also help disseminate the trial’s results beyond the academic and health-care professional community to other patient groups and the wider public.

### Trial management and oversight

**Figure 3:**
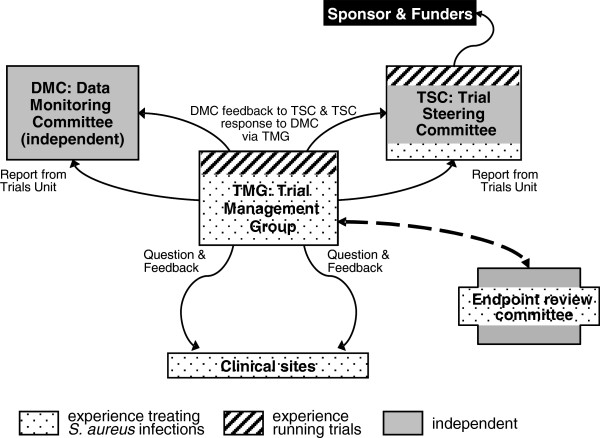
Trial organogram.

The Trial Management Group (TMG) will comprise the trial team at the MRC CTU, plus the Chief Investigator, the principal investigator at each site and the leads of the integral substudies. A Trial Steering Committee (TSC) with a majority of independent members will be responsible for trial oversight. It will be chaired by Dr. Adrian Martineau and will also include an independent microbiologist (Dr. Geoff Scott), an independent trialist and infection specialist (Prof. Jeremy Farrar), and a patient representative from SURF (Mrs. Bostock). The CI (Guy Thwaites) and two other site clinicians (Drs. Hopkins and Barlow) will also be formal voting members of the TSC, although all site investigators may attend TSC meetings as observers. The TSC will meet annually and will have a TSC Charter agreed by all members before the trial starts. The function of these committees and their relationships are expressed in Figure 3.

An independent data-monitoring committee (DMC) will be established to monitor the trial, reviewing trial data on recruitment, safety, adherence to randomised strategies and efficacy, as well as considering findings from any other relevant studies. It will be chaired by Prof. David Lalloo, an infectious diseases specialist, and include an independent microbiologist (Prof. Mark Wilcox), and an independent statistician (Prof. Doug Altman).

### Ancillary studies

There will be two ancillary studies to the main trial:

1. A population pharmacokinetic (PK) and pharmacodynamic (PD) study of rifampicin, flucloxacillin and vancomycin for the treatment of *S. aureus* bacteraemia. Our aim is to determine the pharmacological parameters of rifampicin, flucloxacillin and vancomycin that best predict treatment success and provide a rational basis from which the optimal dose, frequency and route of administration can be modelled statistically and/or explored in future studies. Samples will be taken without knowledge of randomised allocation, although rifampicin concentrations will only be measured in those receiving active drug. The specimens will be batched and the assays performed after every patient in the batch has completed the trial; the laboratory team performing the assays will only be given the treatment codes for each batch of patients and will perform them without knowledge of the patients’ outcomes. No information about rifampicin levels will be available outside the laboratory team before the end of the trial, although these results would be provided to the trial Data Monitoring Committee.

2. The influence of host and bacterial genetics on disease severity and outcome from *S. aureus* bacteraemia. Our aim is to identify host and bacterial genetic factors that influence disease severity (for example, the development of metastatic complications) and poor outcome from *S. aureus* bacteraemia.

### Funding

The main trial is supported by grant funding from the National Institutes of Health Research (NIHR) Health Technology Assessment (HTA) Programme (10/104/25). The ancillary studies are supported by local funding.

## Discussion

The results of the meta-analysis of randomised controlled trial data, together with the findings of observational studies, indicate adjunctive rifampicin may have a surprising and substantial impact on survival from SAB. These data do not, however, constitute evidence of sufficient rigour to influence current treatment guidelines, clinical practice or indeed the equipoise of clinicians recruiting patients into the proposed trial – even clinicians in centres using rifampicin in a greater proportion of patients have indicated their willingness to randomise as they recognise the lack of evidence supporting their practice. In particular, whilst statistically significant, the results from the trial meta-analysis are not convincing as they are based on a small number of patients in a small number of trials over a wide period of time. In addition, the potential negative impacts of rifampicin toxicity, interactions and, in particular, resistance cannot be reliably assessed in these studies. There is, therefore, clear justification for a large randomised controlled trial of adjunctive rifampicin for SAB so that the potential benefits of rifampicin can be evaluated directly and reliably against the potential risks.

The trial addresses the hypothesis that early, enhanced bacterial killing by rifampicin improves the outcome from SAB. Two biomedical substudies are therefore planned to understand the reasons why adjunctive rifampicin either works or fails to work, and how bacterial and host genetic variation influences clinical outcome; both provide the potential for personalising treatment in the future. Demonstrating mechanisms underlying trial results has two major impacts: first, saliency promotes uptake of positive trial results into clinical practice and guidelines and provides reasons for clinicians to stop using ineffective interventions; second, understanding such mechanisms is critical to the rational choice of further interventions to improve patient outcome. Such studies can be conducted to a very high standard when integrated into a large randomised controlled trial, substantially increasing the value of the research.

One of the key considerations when designing this trial was whether to perform an open label or blinded placebo-controlled trial. The argument for an open trial rests on two assumptions: first, it will be impossible to blind patients and physicians, because taking rifampicin turns urine orange, an effect that cannot be replicated by placebo; second, knowing treatment allocation will not matter if the primary endpoints are objectively ascertained (e.g. death). An open trial, however, would run a much higher risk that patients in the control arm would be managed differently than those in the rifampicin arm. In particular, knowledge of the treatment allocation may encourage the attending physicians to prescribe other antibiotics (aminoglycosides, for example) to those in the control arm perceived to be failing treatment. If this were to occur too often, the effect of rifampicin could be obscured by the additional therapy received in the control arm. In addition, physicians may be tempted to investigate the treatment arms differently, perhaps having a lower threshold for ordering radiological investigations in the control arm, changing the likelihood that secondary complications and treatment failure might be detected in these patients.

It is impossible to perform a true ‘double blind’ study using rifampicin, but we believe a blinded study is preferable to an open study. We believe it will be much harder for physicians to predict treatment allocation than might otherwise be presumed. The placebo cannot replicate the urine colouration, but its use will mean treating clinicians will be uncertain of treatment allocation and will, therefore, be much less likely to manage patients differently in the respective treatment arms. There is considerable inter- and intra-individual variability in rifampicin’s effect on urine colour. In some patients, the colouration is slight and can be hard to distinguish from the dark, concentrated urine frequently observed in acutely unwell patients. In many, the orange urine colour becomes less marked over time. In addition, the opportunity for physicians to examine the urine at the bedside will only occur in patients with urinary catheters. Catheters will not be required by all patients and are usually removed at the earliest opportunity. We will also limit the opportunity for physicians to inspect urine by ensuring the catheter bags are emptied regularly and urine it is not allowed to accumulate in large volumes.

There is also an important sociological component to the use of a placebo, which is particularly pertinent in this trial because the patients’ primary physician is likely to be a non-infection specialist (e.g. renal, cardiothoracic). In an open trial, whether or not the patient is receiving rifampicin will be one of the first questions that will be asked when patient management is reviewed (when the infection specialist may not be present) and it is very likely that subsequent management will depend on the answer. Blinding, even if not 100% successful, sends a clear signal that whatever the primary physician believes the patient might be receiving, this should not be taken into account in subsequent management.

Finally, the completed trial will stand alone as being the first adequately powered randomised controlled trial ever performed to address the optimal management of SAB. The results will be scrutinised worldwide and should have a major influence on treatment guidelines within the NHS and beyond. An open trial may compromise the trial’s impact. If the results are negative, critics may point to the possibility, detailed above, that physicians treated control patients differently and rifampicin’s effect was disguised. Likewise, if the results are positive some may question whether knowledge of the treatment allocation led to enhanced care in the rifampicin arm. It is important to foresee how the trial’s results may be interpreted and we believe that an open label trial may compromise the potential impact of the trial and thereby limit the value of the research.

### Trial status

The ARREST trial was conceived and designed by members of the UKCIRG in 2010, with successful application for peer-reviewed funding by the UK National Institute for Health Research Health (NIHR) Health Technology Assessment (HTA) Board in November 2011 (project no. 10/104/25). The trial will start recruitment in December 2012 and will take 3 years to complete; it will be managed through the Medical Research Council Clinical Trials Unit (MRC CTU) in London. The full version of the current trial protocol is available at: http://www.ctu.mrc.ac.uk/research_areas/study_details.aspx?s=262

## Competing interests

All the authors declare that they have no competing interests.

## Authors’ contributions

The research question was identified by GT with consultation with all members of the UKCIRG. The ARREST trial protocol was drafted by GT and ASW with comment and revision by all members of the UKCIRG. GT was responsible for drafting this paper, but all authors provided comments and review of the drafts and approved the final version. The views and opinions expressed in this publication are those of the authors and do not necessarily reflect those of the National Health Service, the NIHR, the NIHR Evaluation, Trials and Studies Coordinating Centre, the HTA programme or the Department of Health. All authors read and approved the final manuscript.
